# A122 MINIMALLY INVASIVE ENDOSCOPIC RESECTION TECHNIQUES FOR ANORECTAL JUNCTION NEOPLASIA: A SYSTEMATIC REVIEW AND META-ANALYSIS

**DOI:** 10.1093/jcag/gwac036.122

**Published:** 2023-03-07

**Authors:** A A Arif, K Donaldson, H Qian, E Lam, N Shahidi

**Affiliations:** 1 University of British Columbia; 2 Centre for Health Evaluation and Outcome Sciences; 3 St. Paul's Hospital , Vancouver, Canada

## Abstract

**Background:**

The management of neoplastic lesions at the anorectal junction remains debated. Endoscopic submucosal dissection (ESD) and endoscopic mucosal resection (EMR) have emerged as the primary endoscopic modalities of choice.

**Purpose:**

We sought to compare the performance of ESD and EMR in resection of anorectal neoplasia.

**Method:**

Two authors independently searched MEDLINE, EMBASE and Cochrane Libraries (Jan 2000 – Aug 2021) for citations evaluating the performance of endoscopic resection techniques (ESD, EMR) for lesions involving the anorectal junction (defined as within 20mm of the dentate line). The frequencies and 95% confidence intervals (95% CI) of technical success (complete removal of all neoplastic tissue at index procedure), clinically significant post-endoscopic resection bleeding (CSPEB), delayed perforation, recurrence and referral to surgery were assessed using random-effects modelling.

**Result(s):**

We included 11 studies (total 563 patients: 414 ESD, 149 EMR) of which nine were ESD and two were EMR studies. Technical success was achieved in 97.2% overall (95% CI 94.8%-98.5%, ESD 97.5% and EMR range 93.9%-98.0%). Clinically significant post-endoscopic resection bleeding occurred in 4.3% (95% CI 1.6%-11.1%, ESD 3.0% and EMR range 8.2%-11.0%). Delayed perforation was not identified. Recurrence at first screening colonoscopy occurred in 4.8% (95% CI 1.9%-11.7%, ESD 3.0% and EMR range 15.4%-18.4%). Referral to surgery for any reason occurred in 5.9% (95% CI 4.3%-8.0%, ESD 6.9%, EMR range 2.0%-3.0%).

**Conclusion(s):**

ESD and EMR demonstrate high frequencies of technical success but may have different rates of adverse events and recurrence. More studies investigating lesions at the anorectal junction should be conducted including head-to-head analyses between ESD and EMR for low-risk anorectal junction neoplasia.

**Please acknowledge all funding agencies by checking the applicable boxes below:**

None

**Disclosure of Interest:**

None Declared

